# Risk Factors for Burnout Among Doctors in a Tertiary General Hospital

**DOI:** 10.1192/bjo.2024.194

**Published:** 2024-08-01

**Authors:** Leslie Lim, Louis Loh, Yiong-Huak Chan, Leonard Eng, Johnson Fam

**Affiliations:** 1 Singapore General Hospital, Singapore, Singapore; 2 National University of Singapore L University, Singapore, Singapore

## Abstract

**Aims:**

To study the risk factors for burnout among doctors in a tertiary general hospital in Singapore. We hypothesized that burnout would be associated with singles, young age, females, foreign born staff who had recently moved to this country unaccompanied by family, and those showing less resilience. We hypothesised perceived support and satisfaction with leisure would mitigate against burnout.

**Methods:**

An anonymised survey was carried out, with questionnaires sent to all staff via email. Survey instruments included the Oldenburg Burnout Inventory, Connor Davidson Resilience Scale, Brief Form of Perceived Social Support Questionnaire, Patient Health Questionnaire-4 items (PHQ-4), Leisure Time Satisfaction Survey and the Demand Control Support Questionnaire (DCSQ). Descriptive statistics for normally-distributed numerical variables were presented as mean (SD or standard deviation), and for categorical variables, median and n (%). One-way ANOVA was performed to determine differences in total burnout scores across categorical variables and simple linear regression was used to assess for binary and numerical outcomes in terms of resilience, PHQ, DSCQ, satisfaction with leisure time and perceived social support, with significance set as p < 0.05.

**Results:**

A total of 129 doctors responded to the survey. Over half were male, and nearly 70% were married. Nearly half were below age 40 and only about 5% had no immediate family living in Singapore.

Burnout was associated with young age (p < 0.004) and those with anxiety 2.39 (2.13 to 2.64) p = 0.038, and depressive symptoms 2.71 (2.44 to 2.97) p < 0.001. Psychological demand was positively associated with burnout (1.52 (1.32 to 1.71) p < 0.001; whereas decision latitude −0.69 (−0.85 to −0.52), social support at work −1.35 (−1.49 to −1.21), and high resilience −0.56 (−0.63 to −0.48), were negatively associated (all p < 0.001).

Satisfaction with leisure time was negatively correlated with burnout (p < 0.001). Contrary to hypothesis, singlehood, gender, overseas staff recently joined with no accompanying family were not associated with burnout (p > 0.05). In addition, perceived social support from outside work did not mitigate against burnout (p > 0.05).

**Conclusion:**

Young age, anxiety and depression, and psychological demands were risk factors, whereas resilience, decision latitude, satisfaction with leisure, and social support at work were protective factors against burnout. Reducing workload, improving work schedules, promoting self-management, teaching physical, mental, and emotional self-care, and other stress management activities are among the effective techniques shown to reduce burnout. Interventions should be made available for all staff, but specifically focusing on those at greatest risk.

